# Automated Text Messaging With Patients in Department of Veterans Affairs Specialty Clinics: Cluster Randomized Trial

**DOI:** 10.2196/14750

**Published:** 2019-08-04

**Authors:** Vera Yakovchenko, Timothy P Hogan, Thomas K Houston, Lorilei Richardson, Jessica Lipschitz, Beth Ann Petrakis, Chris Gillespie, D Keith McInnes

**Affiliations:** 1 Center for Healthcare Organization and Implementation Research Bedford Department of Veterans Affairs Medical Center Department of Veterans Affairs Bedford, MA United States; 2 Division of Health Informatics and Implementation Science Department of Population and Quantitative Health Sciences University of Massachusetts Medical School Worcester, MA United States; 3 Division of Health Informatics and Implementation Science Department of Population and Quantitative Health Sciences University of Massachusetts Medical School Amherst, MA United States; 4 Brigham and Women’s Hospital Department of Psychiatry Boston, MA United States; 5 Harvard Medical School Boston, MA United States; 6 Department of Health Law Policy and Management Boston University School of Public Health Boston, MA United States

**Keywords:** implementation facilitation, texting, veterans, eHealth, self-management, digital health, digital medicine

## Abstract

**Background:**

Acceptability of mobile phone text messaging as a means of asynchronous communication between health care systems and patients is growing. The US Department of Veterans Affairs (VA) has adopted an automated texting system (aTS) for national rollout. The aTS allows providers to develop clinical texting protocols to promote patient self-management and allows clinical teams to monitor patient progress between in-person visits. Texting-supported hepatitis C virus (HCV) treatment has not been previously tested.

**Objective:**

Guided by the Practical, Robust Implementation and Sustainability Model (PRISM), we developed an aTS HCV protocol and conducted a mixed methods, hybrid type 2 effectiveness implementation study comparing two programs supporting implementation of the aTS HCV protocol for medication adherence in patients with HCV.

**Methods:**

Seven VA HCV specialty clinics were randomized to usual aTS implementation versus an augmented implementation facilitation program. Implementation process measures included facilitation metrics, usability, and usefulness. Implementation outcomes included provider and patient use of the aTS HCV protocol, and effectiveness outcomes included medication adherence, health perceptions and behaviors, and sustained virologic response (SVR).

**Results:**

Across the seven randomized clinics, there were 293 facilitation events using a core set of nine implementation strategies (157 events in augmented implementation facilitation, 136 events in usual implementation). Providers found the aTS appropriate with high potential for scale-up but not without difficulties in startup, patient selection and recruitment, and clinic workflow integration. Patients largely found the aTS easy to use and helpful; however, low perceived need for self-management support contributed to high declination. Reach and use was modest with 197 patients approached, 71 (36%) enrolled, 50 (25%) authenticated, and 32 (16%) using the aTS. In augmented implementation facilitation clinics, more patients actively used the aTS HCV protocol compared with usual clinic patients (20% vs 12%). Patients who texted reported lower distress about failing HCV treatment (13/15, 87%, vs 8/15, 53%; *P*=.05) and better adherence to HCV medication (11/15, 73%, reporting excellent adherence vs 6/15, 40%; *P*=.06), although SVR did not differ by group.

**Conclusions:**

The aTS is a promising intervention for improving patient self-management; however, augmented approaches to implementation may be needed to support clinician buy-in and patient engagement. Considering the behavioral, social, organizational, and technical scale-up challenges that we documented, successful and sustained implementation of the aTS may require implementation strategies that operate at the clinic, provider, and patient levels.

**Trial Registration:**

Retrospectively registered at ClinicalTrials.gov NCT03898349; https://clinicaltrials.gov/ct2/show/NCT03898349

## Introduction

Short message service (SMS or texting) is becoming an accepted means of asynchronous communication between health care systems and patients, supporting appointment attendance, medication taking, and medication refill reminders [1]. Texting interventions have been studied across a range of clinical domains and stages of care [2]. Despite the ubiquity of cell phones and the established benefits of texting interventions, there has been limited research on the implementation of patient texting in health care systems [2]. Although texting interventions have yielded improvements in processes of care and health outcomes, results have been achieved through heterogenous approaches, revealing significant implementation knowledge gaps [3-5]. Furthermore, technologies intended to directly engage patients (patient-facing technologies) encounter distinct implementation challenges when clinical staff are needed to promote patients’ adoption of the technology.

Implementation facilitation, a kind of meta-strategy composed of multiple implementation strategies, uses experts in clinical, process, and implementation issues to solve problems and offer support that enables others to institute and sustain practice change [6-8]. Although facilitation is versatile, it has not been extensively studied as part of health information technology implementation or sustainability efforts [9,10].

In 2016, the US Department of Veterans Affairs (VA) began piloting an automated text messaging system (aTS) for patient self-management modeled after texting systems used in the UK National Health Service (NHS), Australia, and Canada [11,12]. The aTS, titled Annie after Annie Fox, RN, the first nurse to be awarded a Purple Heart, provides patients with a technology to become more engaged in their own care through condition-specific protocols to accentuate the key points of care plans. The aTS can send one-way messages and interpret patient messages following specified syntax and then reply through rule-based logic with two-way (bidirectional) messages.

The rollout of VA’s aTS presented a unique opportunity to examine implementation in the context of a large, integrated health care system. We chose to evaluate the implementation and effectiveness of the aTS across specialty care hepatitis C virus (HCV) clinics and test the utility of implementation facilitation. We focused exclusively on HCV treatment because it involves a time-limited predefined course of daily medication, and high adherence is required to achieve sustained virologic response (SVR) and avoid drug resistance [13,14]. Adherence to follow-up appointments and regular bloodwork are also essential to successful treatment.

While there is ample evidence regarding the effectiveness of texting to support medication taking in other disease contexts, no studies, to our knowledge, have examined HCV treatment support via texting [2]. Given the evidence for similar conditions, we hypothesized that texting for HCV would have comparable effects. As such, we conducted a hybrid type 2 implementation study to simultaneously examine both clinical and implementation outcomes and thus generate evidence in this area [15]. Our aims were to (1) qualitatively and quantitatively assess implementation outcomes for the aTS and (2) assess impact of the aTS on HCV clinical outcomes in a real-world setting.

## Methods

### Design

This was a multisite, mixed methods, randomized, two-group hybrid type 2 study design comparing the effectiveness of usual implementation (UI) and augmented implementation (AI) facilitation. Matched comparison (ie, no intervention) sites helped to determine effectiveness of the aTS in aiding HCV treatment. The study was reviewed by the institutional review board at the Edith Nourse Rogers Memorial Veterans Hospital in Bedford, Massachusetts, and determined to be a quality improvement study and therefore exempt (VA Handbook 1058.05) [16]. The project was conducted from February 2017 through February 2018. Due to an error, this study was registered retrospectively. The study was registered at ClinicalTrials.gov (NCT03898349).

### Setting and Participants

Nine HCV clinics participated in this study. Seven served as intervention sites and two as matched comparisons. The group of clinics selected reflects a purposive sample based on criteria including clinic size and complexity as well as geography. HCV clinics were recruited via a national HCV provider email listserv and monthly HCV provider phone call. The seven HCV intervention clinics selected were randomly assigned to either UI or AI, using set randomization, which, with small sample sizes, helps to achieve balance on a set of relevant characteristics—in this case, urban/suburban setting and HCV patient volume [17]. Two additional comparison clinics were selected purposively because they had similar patient volume, clinic complexity, and geographic locale to the other participating clinics [18]. In total, data were collected from nine VA clinics: four AI clinics, three UI clinics, and two comparison clinics. Care teams within the HCV clinics had different compositions and involved pharmacists, nurse practitioners, registered nurses, and social worker to varying degrees.

### Text Messaging Protocol for Hepatitis C Virus Treatment

At the time of implementation, usual care for HCV included starting a patient on daily oral medication for 8 to 16 weeks with follow-up in-person visits, blood lab work, and medication refills at 2- or 4-week intervals. Using the aTS in the context of HCV was intended to improve processes, outcomes of care, and satisfaction with care. The HCV texting protocol included reminder text messages for each modifiable behavior in the HCV treatment process: medication taking, appointment attendance, laboratory completion, and self-efficacy to encourage continued engagement in treatment. Typically, clinic providers and staff would reach out by phone or letter to remind patients of HCV appointments, labs, and refills. To ensure alignment with standard treatment processes and local clinic workflow, each of the seven clinics co-designed and tailored the HCV texting protocol (eg, adjusting messaging logic from 2 to 4 weeks for different treatment intervals) in conjunction with study team members (VY, KM, and national aTS program office technical and clinical specialists). Motivational messages were written to be supportive in nature, promote self-management, and increase feelings of connection to the treatment team. Principles of universal design were also incorporated to ensure that different ranges of abilities, access, and equity were considered and the widest reach and benefit of the HCV texting protocol could be achieved [19]. Sample HCV texting protocol messages are as follows:

- Medication reminder: “Hi, it’s Annie, with a helpful reminder. Did you remember to take your HepC medication today?”
- Appointment reminder: “Hi, Annie here. Don’t forget about your upcoming HepC appointment. If you do not know when your appointment is, please call to find out.”
- Lab reminder: “Annie & VA Liver team here. You are due to have a blood draw this week so we can see how your HepC meds are working. Please call your VA care team if you need help getting your labs.”
- Motivational message: “Don’t forget that the HepC Team is here to support you in your HepC treatment efforts. Call your care team if we can help you. – Annie”

Messages could be tailored for content (eg, adding clinic name, phone number, or appointment date) and timing (eg, adjusting time of day that medication reminder is sent) through patient and provider discussion at the time of aTS enrollment or later to reflect patient preferences. Veterans who did not use the aTS received otherwise standard HCV care.

### Usual Implementation and Augmented Implementation Facilitation

Facilitation was employed to support adoption of the aTS at participating clinics, with facilitation delivered by a primary (VY) and a secondary (KM) external facilitator. During the 4-month preimplementation phase, the functions of the external facilitators included engaging local, regional, and national stakeholders to garner support for the aTS. The implementation phase took place over 6 months and differed between UI and AI. The postimplementation (evaluation) phase took place over 3 months.

### Usual Implementation Clinics

UI clinics received the start-up experience that VA designed for all new clinics instituting the aTS. This involved a live virtual demonstration of the aTS and access to an aTS resource website that included promotional materials and training guides. UI clinics could receive troubleshooting assistance from the external facilitators by phone or email but only if and when they reached out to the them.

### Augmented Implementation Clinics

In addition to the start-up experience for UI sites, AI clinics received an implementation toolkit, support for local champion development, and proactive outreach by the primary external facilitator. The toolkit was developed by our team based on a formative evaluation that involved visits to five VA medical centers around the country that were using a pilot version of the aTS for conditions other than HCV. The toolkit contained sections on evidence of texting in health care, suggestions for gaining leadership and clinic support for technology like the aTS, use of champions to support aTS adoption, tips and tools on how to use the aTS, and aTS promotional materials to encourage clinic and patient participation. Each AI clinic received one in-person visit from the primary external facilitator early in their implementation efforts. Additionally, the primary external facilitator initiated check-ins with AI clinic champions throughout implementation.

Facilitation was delivered via email, phone, and in person. In the preimplementation period, to establish rapport and trust, there was more emphasis on phone calls and in-person meetings, whereas during implementation, those modes were used less while use of emails increased. Facilitation calls lasted from 5 minutes to 90 minutes (40-minute average) and site visits by the external facilitator lasted 2 to 4 hours. The facilitation meta-strategies included assessing for readiness to implement, site visits, identifying and preparing champions, developing and distributing educational materials, building a coalition, local technical assistance, and tailoring implementation to context [20,21].

### Conceptual Framework

Our evaluation was guided by the Practical, Robust Implementation and Sustainability Model (PRISM), which defines a set of factors for consideration when designing, implementing, sustaining, and evaluating interventions [22]. PRISM posits that the extent to which an intervention achieves results can be linked to the four PRISM domains: intervention characteristics (via patient and organizational perspectives), intervention recipients (via patient and organizational perspectives), external environment, and implementation and sustainability infrastructure.

### Measures and Data Collection

PRISM domains (intervention characteristics, recipients via patient and organizational perspectives, external environment, and implementation and sustainability infrastructure) guided the measures and data collection and are denoted in parentheticals.

#### Implementation Processes: Facilitation (Implementation and Sustainability Infrastructure)

The primary external facilitator logged facilitation events on a tracking sheet, including facilitation date, length of time, mode of delivery (ie, email, phone call, or in-person visit), purpose, notes, and other observations. If multiple facilitation events occurred in one day, only one event per person per day was counted.

#### Implementation Outcomes: Texting Use (Intervention)

Providers logged the number of patients who were offered the aTS and noted whether patients enrolled or declined, including the reason for declining. The content of patient text message replies was extracted from the aTS portal. To be eligible for the texting protocol, patients had to be starting HCV medication treatment during the implementation period. There were four steps to initiate a patient on the aTS: (1) providers verbally offered patients the aTS, (2) providers registered interested patients in the aTS portal and assigned them the HCV protocol, (3) once a patient was registered, the aTS would send an automated text message requesting the patient authenticate themselves by replying to this initial text message thus prompting the assigned HCV protocol to begin, and (4) patients actively texted with the aTS.

#### Clinical Effectiveness Outcomes

Medication adherence was measured via patient text response rate, operationalized as the number of days of text-confirmed medication taking divided by the number of days receiving medication reminder texts. Consistent with other adherence standards, an affirmative text response rate of ≥80% was considered high adherence [23]. Clinical data, including HCV treatment regimen and duration and lab results, were extracted from VA’s national HCV dashboard based on data from VA corporate data warehouse. The goal of HCV treatment is to achieve cure or SVR, meaning there is an undetectable HCV lab result 12 weeks after completion of treatment.

#### Questionnaires (Recipients, Intervention, Implementation)

Patients at each clinic completed baseline and follow-up (after 8 to 12 weeks of using the aTS) questionnaires. The comparison clinics followed the same schedule, although without any use of texting. Patient questionnaires covered the topics of self-rated health status, adherence, illness perception, health engagement and activation, technology use, experiences with the aTS (usability, usefulness, working alliance), and demographics [24-29]. Provider questionnaires followed the same schedule and covered topics of technology experience, quality improvement culture, climate and readiness for implementation, satisfaction with current local HCV care processes, experiences with the aTS (usability and usefulness), and demographics [30-33]. Questionnaires were pretested for clarity, redundancy, and relevancy by two patients and two providers and two implementation scientists independent of the study team.

#### Semistructured Interviews (All Practical, Robust Implementation and Sustainability Model Domains)

Qualitative semistructured telephone interviews were conducted with patients and providers who used or were invited to use the aTS. Interviews were conducted in the follow-up period during September and October 2017. The interview guides were informed by PRISM domains and explored issues regarding barriers and facilitators to aTS uptake and use (intervention, implementation, and sustainability), usability and usefulness of the aTS (intervention), and how the aTS was experienced by patients and providers (recipients) in the course of treatment and daily practice (external environment). Interviews were conducted by members of the study team not involved in facilitation (BP, CG) and lasted about 30 minutes.

**Figure 1:**
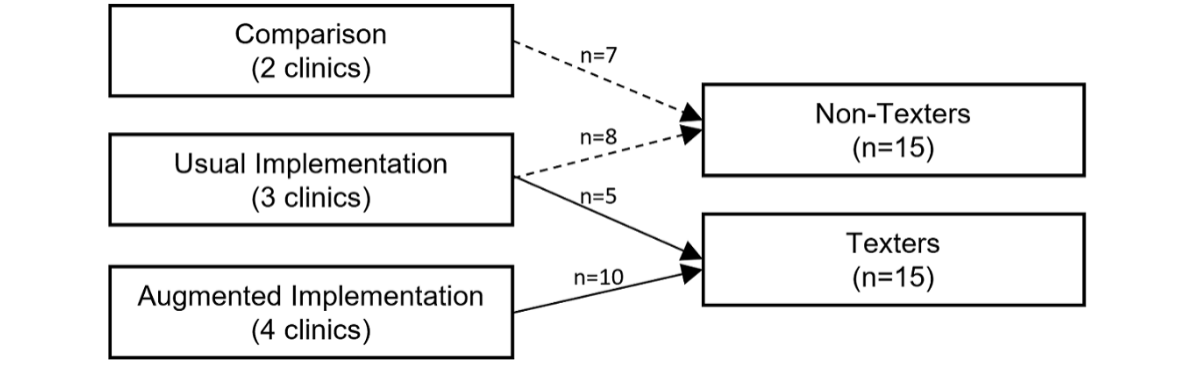
Flow diagram of clinics in the study and participants, identified as texters and nontexters.

Due to the small number of participants who successfully used the aTS, it was decided that comparing texters, regardless of group (AI or UI), against nontexters was necessary. For effectiveness outcomes measures, we combined patients who were using the aTS regardless of whether they were in UI or AI clinics. These were referred to as texters. In contrast, nontexters were defined as patients who agreed to participate in the project but never completed the step of authenticating themselves with the aTS (at either UI or AI clinics), and thus never received any text messages, as well as patients from the two comparison clinics that did not implement the aTS (Figure 1). For each questionnaire or qualitative interview completed, patients received a $10 store gift card to compensate them for their time.

### Data Analysis

Descriptive and bivariate analyses of facilitation log data were conducted to compare facilitation dose between UI and AI groups. Descriptive and bivariate analyses were conducted on provider and patient questionnaires, text message frequencies, and clinical data to assess differences between implementation groups (UI and AI) in implementation outcomes. We then compared clinical effectiveness outcomes between texters and nontexters. We examined patient progression through the aTS initiation process by calculating the percentage retained from one step to the next by UI and AI group. Chi-square tests were used to assess differences between the two groups. All analyses were conducted in RStudio version 1.0.153 (The R Foundation), and statistical significance was defined as *P*<.05.

Qualitative interviews were audio recorded, transcribed verbatim, and analyzed using NVivo 11 (QSR International Pty Ltd) software. Thematic analysis of all qualitative data (interview transcripts, facilitator meeting notes, text messages) was conducted [34]. PRISM domains provided deductive a priori codes and other codes emerged through inductive coding. The triangulation of quantitative and qualitative data served as the final step of analysis.

## Results

Findings are arranged by relevant PRISM domain (intervention characteristics, recipients via both patient and organizational perspectives, external environment, and implementation and sustainability infrastructure).

### Recipient Perspectives and Organizational Characteristics

Of the nine HCV clinics, seven were in the northeastern United States (including the two comparison clinics), and two were in the western United States (one each AI and UI). In total, fifteen providers across the intervention clinics (seven in UI and eight in AI) were trained to use the aTS and completed a baseline demographic questionnaire. Ten of these providers eventually enrolled patients and completed a follow-up questionnaire (five providers were unable to enroll any patients). At baseline, clinic and provider characteristics were balanced on age, sex, and technology experience (data not shown) across AI, UI, and comparison clinics. Provider surveys indicated there were no differences between clinics in the two implementation arms on readiness to implement the aTS, including on measures of perceived evidence strength for texting, organizational context, and implementation climate (data not shown). These surveys indicated there were, however, differences in satisfaction with clinic HCV treatment practices: 100% (7/7) of UI compared with 50% (4/8) of AI providers were satisfied with their local HCV treatment processes (*P*=.04).

### External Environment

Providers were generally eager to support HCV treatment with the aTS because improving HCV treatment was a national and local VA priority. One UI provider mentioned that their clinic had already been considering creating a texting reminder system:

We’re actually really excited to use it [the aTS] with our patients. This is something that we had talked about doing or developing something like this to see what the impact could be on improving adherence to appointments, adherence to medications for patients.

Another provider lamented:

I wish this [the aTS] could have come earlier.

### Implementation Facilitation Processes

**Figure 2:**
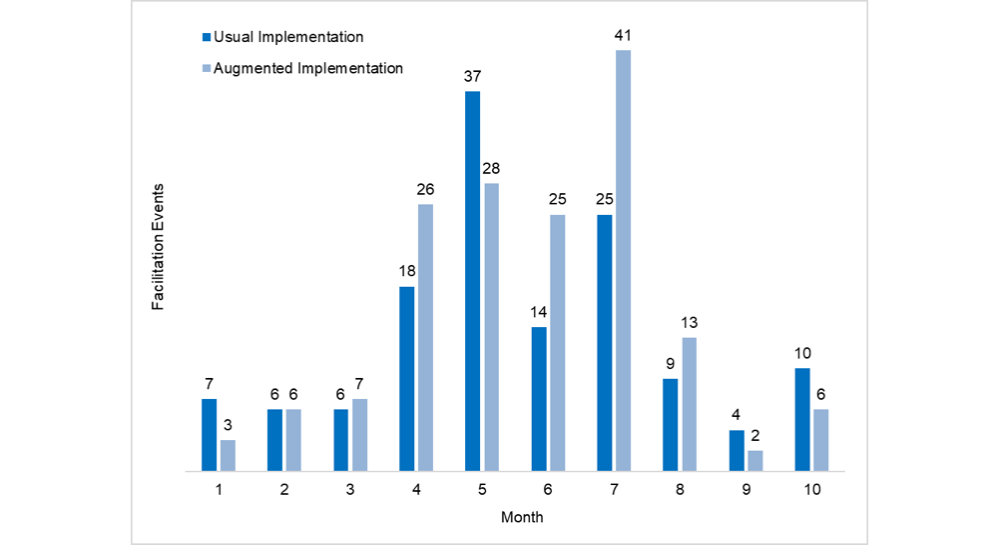
Facilitation events over time by clinic and implementation arm.

Figure 2 summarizes email, phone, and in-person facilitation events (n=293) across the seven intervention clinics. Facilitation effort was relatively modest, initially, as new clinics were adopting and learning the aTS, rising to the busiest period in months 4 to 7 and tapering off in months 8 to 10. AI clinics had an average of 39 facilitation events compared with 45 for UI clinics (*P*=.17), or about weekly contact. Only 10% of providers reported (via postimplementation survey) that they could have implemented the aTS without facilitation. The association between facilitation dose and provider-initiated aTS recruitment was positive and linear, although not statistically significant (*r*=.71, *P*=.07).

Providers across implementation arms had largely positive feedback about the facilitation received to support aTS implementation. One provider explained the value of an accessible facilitator:

...whenever you’re using new technology and new approaches with the technology component, it’s just good to have somebody that you can, who’s very responsive...and can find out the answer for you in a timely fashion.

In the case of AI clinics, providers highlighted the value of the in-person site visit because “when [the facilitator] came it kind of clinched it,” suggesting the one-on-one visit helped providers make the decision to use the aTS and provided an important opportunity to ask clarifying questions and cement more of the technical and logistical aspects of implementation. AI clinic providers had mixed impressions of the toolkit. Providers felt it provided needed information and was easy to understand; however, some felt it was too long and overly dense. Several suggested that an abridged quick start guide would have been more useful.

### Implementation Outcomes

**Figure 3:**
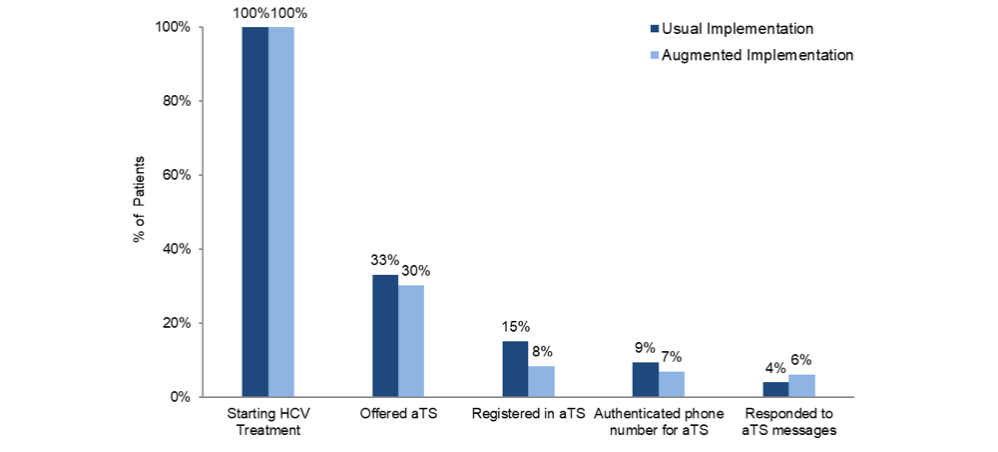
Texting engagement by implementation and facilitation arm. HCV: hepatitis C virus; aTS: automated texting system.

Implementation outcomes, UI versus AI, are depicted in the retention diagram in Figure 3, which shows the percentage of patients approached to participate in the aTS who progressed from one step to the next. Across the seven aTS intervention clinics, a total of 625 patients started HCV treatment during the implementation period. Providers comparably offered the aTS to 33% (92/278) of patients at UI sites and 30% (105/347) of patients at AI sites who were starting HCV treatment (*P*=.45). Notably, UI sites did better in the registration and authentication steps, and AI sites did better in reaching the final step of aTS message interaction. By implementation group there was a significant difference in patient registration (42/278, 15%, UI vs 29/347, 8%, AI; *P*=.01) and a borderline significant difference in patient authentication (26/278, 9%, UI vs 24/347, 7%, AI; *P*=.06). However, compared to UI site patients, a greater percentage of AI site patients who authenticated their phone numbers went on to actively respond to HCV messages (11/278, 4%, UI vs 21/347, 6%, AI; *P*<.001). Of 35 patients (14 UI vs 21 AI) receiving medication reminder text messages, 91% (32/35) replied to at least one medication reminder. The mean medication reminder response rate was 78% (SD 26%; median 89%), with no difference between groups (77% UI vs 79% AI; *P*=.87).

Qualitative interviews with providers indicated that they were often choosing to offer the aTS to younger and clinically less complex candidate patients who they perceived to be more technologically savvy and thus more likely to agree to use the system. In correlation analysis, the number of patients to whom the aTS was offered was inversely associated with providers’ baseline satisfaction with HCV care process (*r*=–0.65, *P*=.06) and their length of time working in the VA health care system (*r*=–0.78, *P*=.01). Nevertheless, all providers were surprised that a high percentage of patients declined the aTS offer:

I thought a lot of people are going to be able to participate, but I guess when we started offering them [the text messages] some patients don’t, I guess they’re not used to it, so they, most of the patients that I offer to decline to participate.

Patient reasons for declining the aTS fell into four categories: general disinterest (48%), texting apprehension, including cost concerns (30%), beliefs of already being good at medication adherence (12%), or beliefs that texting would duplicate other self-management approaches (10%).

### Patient Characteristics

There were few demographic differences between texters and nontexters (Table 1), although texters were more likely to be black (13/15, 87%, vs 6/15, 40%; *P*=.02) and less likely to have a self-reported mental health or substance use disorder (9/15, 60%, vs 14/15, 93%; *P*=.03).

### Clinical Effectiveness Outcomes

On the follow-up survey (Table 2), more texters versus nontexters reported excellent health (4/15, 27%, vs 0/15, 0%; *P*=.01) and greater ability to prevent or reduce problems associated with their health (9/15, 60%, vs 2/15, 13%; *P*=.03). A greater proportion of texters compared with nontexters had overall less negative HCV illness perception and reported fewer concerns about failing HCV treatment (13/15, 87%, vs 8/15, 53%, respectively; *P*=.05). Texters reported better or about equal HCV treatment adherence than nontexters, including having higher perceptions of their own HCV adherence than did nontexters (11/15, 73%, reporting excellent adherence vs 6/15, 40%; *P*=.06). For patients for whom lab results were available, 96% (27/28) of texters had achieved SVR compared with 94% (153/163) of nontexters (*P*>.99), reflecting the high cure rates possible with current HCV treatments and our small sample size.

**Table 1:** Patient characteristics by automated test messaging system use at follow-up (n=30).

Characteristic		Nontexters^a^ (n=15)	Texters^b^ (n=15)	*P* value
Age in years, mean (SD)		62 (11)	62 (5)	.56
Gender (male), n (%)		14 (94)	14 (94)	>.99
**Race, n (%)**				
	African American or black	6 (40)	13 (87)	—
	White	8 (53)	2 (13)	—
	Other	1 (7)	0 (0)	—
Hispanic/Latino, n (%)		1 (7)	0 (0)	>.99
**Marital status, n (%)**				.93
	Single/never married	5 (33)	4 (27)	—
	Married/in a relationship	2 (13)	2 (13)	—
	Divorced/separated	7 (47)	7 (47)	—
	Widowed	1 (7)	2 (13)	—
Employment, currently working, n (%)		2 (13)	3 (20)	.38
**Income, n (%)**				.25
	>$10,000	5 (33)	2 (13)	—
	$10,000 to $20,000	5 (33)	4 (27)	—
	$20,000 to $40,000	4 (27)	4 (27)	—
	<$40,000	1 (7)	5 (33)	—
**Education, n (%)**				.27
	High school or less	9 (60)	5 (33)	—
	Some college or more	6 (40)	10 (67)	—
**Housing, n (%)**				.10
	Own apartment or house	7 (47)	11 (73)	—
	Hospital, domiciliary, shelter, street, drug treatment center	6 (40)	1 (7)	—
	With friend or relative	2 (13)	3 (20)	—
Self-reported mental health or substance use disorder, n (%)		14 (93)	9 (60)	.03
**Social/emotional support**				.61
	Always/usually	3 (20)	8 (53)	—
	Sometime/rarely	12 (80)	7 (47)	—
**Texting history, n (%)**				—
	Unlimited texting plan	10 (67)	11 (73)	.83
	Daily texting	8 (53)	12 (80)	.27

^a^Nontexters: patients who never received any text messages (could be from usual or augmented implementation clinics or comparison clinics).

^b^Texters: patients who used the automated texting system regardless of whether they were in usual or augmented implementation clinics.

**Table 2:** Patient self-reported outcomes by texting group.

Characteristics			Nontexters^a^ (n=15)	Texters^b^ (n=15)	*P* value
**Health status**				
	In general, how would you rate your health (excellent)?, n (%)		0 (0)	4 (27)	.01
	**For how many days during the past 30 days..., n**				
		was your physical health not good?	10	5	.08
		was your mental health not good?	13	8	.23
		did poor physical or mental health keep you from doing your usual activities?	10	5	.25
**Patient activation (strongly agree), n (%)**					
	I am responsible for taking care of my health.		10 (67)	12 (80)	.61
	I am able to prevent or reduce problems associated with my health.		2 (13)	9 (60)	.03
	I can follow through on recommended medical treatment.		5 (33)	10 (67)	.11
**Illness perception (strongly disagree), n (%)**					
	Feeling angry, scared and/or depressed when I think about living with HCV^c^.		4 (27)	7 (47)	.32
	Feeling that HCV controls my life.		5 (33)	8 (53)	.58
	Feeling overwhelmed by the demands of living with HCV.		5 (33)	9 (60)	.21
	Not feeling motivated to go through HCV treatment.		7 (47)	10 (67)	.51
	Feeling that I am failing with my HCV treatment.		8 (53)	13 (87)	.05
**HCV treatment behavior (strongly disagree), n (%)**					
	I forget to take my HCV medicine(s).		11 (73)	12 (80)	>.99
	I decide not to take my HCV medicine(s).		11 (73)	14 (93)	.26
	I forget to get my HCV prescription(s) filled.		12 (80)	11 (73)	.52
	I tend to forget to get my HCV lab and blood work done.		7 (47)	13 (87)	.07
	I run out of my HCV medicine(s).		11 (73)	12 (80)	>.99
	I tend to miss my doctors’ appointments, n (%)		5 (33)	11 (73)	.10
**Self-report HCV medication adherence**					
	Percentage of HCV medication taken correctly in last 4 weeks, mean (SD)		95 (13)	99 (2)	.20
	Ability to take HCV medication as prescribed (excellent), n (%)		6 (40)	11 (73)	.06

^a^Nontexters: patients who never received any text messages (could be from usual or augmented implementation clinics or comparison clinics).

^b^Texters: patients who used the automated texting system regardless of whether they were in usual or augmented implementation clinics.

^c^HCV: hepatitis C virus.

### Intervention Usability, Workflow, and Value

Usefulness and usability of the aTS was assessed with patients and providers. Among those patients using the aTS (texters), 15 completed a follow-up survey and 13 also participated in a semistructured interview. Another 15 patients who did not use the aTS (nontexters) completed a follow-up survey. Among patients, there were no differences by implementation arm (AI vs UI) on patient measures of usability, usefulness, and degree of working alliance with the aTS (data not shown). Texters reported mostly positive sentiments about interacting with the aTS, saying it was easy, simple, and “not rocket science.” Still, some patients struggled with the aTS authentication syntax (“Start”) due to capitalization and punctuation errors (eg, “START” and “start.”). Most (12/15, 80%) preferred text reminders to appointment reminders delivered by mail or phone. About half (8/15, 53%) reported the cost of text messaging could be a barrier to use, although at least 73% (11/15) had an unlimited texting plan. In patients’ opinions, the aTS had benefits in helping stay engaged in care, stay connected to their health care team, and assisting with HCV medication adherence (all 100% positive endorsements). As one patient recognized: “I wouldn’t have been as efficient or effective without some assistance” and it “[gave] me encouragement for doing what I was supposed to.” Most patients (13/15, 87%) were on multiple medications and viewed the aTS as supporting their overall medication-taking routine: “I use [the aTS] for other medications as well...I just group them all in together.” Providers corroborated patient feedback by observing that the aTS “helps relieve a lot of [patient] anxiety about missing a med.”

Provider perspectives on usefulness and usability were captured via follow-up questionnaires and semistructured interviews. Most providers (7/10, 70%) logged into the aTS at least weekly to either assign a protocol to a patient or monitor messages in the aTS dashboard. Some providers tailored protocols according to patient preferences, while other providers opted to retain default protocol settings to streamline the enrollment process. Some indicated that the message history data could be made more usable.

Even with training and ongoing support, providers had startup difficulties with the aTS:

I mean it took me a little while to familiarize myself because the training versus actually doing it yourself, you know, there’s a learning curve...

Half of providers (5/10, 50%) felt that enrolling patients was difficult, and half also felt the aTS did not easily integrate into clinical workflow. One third (3/10, 30%) said using the aTS added a lot of work to their workday. Providers described the aTS as a little bulky, a little cumbersome, and labor intensive. To enhance uptake of the aTS, providers recommended the system become more streamlined and more intuitive, particularly at the stage of registering patients. Providers also suggested integrating the aTS with other VA technologies, including registration kiosks, the electronic medical record, and patient portal (MyHealtheVet).

Although providers saw the aTS as a potential benefit to their practice, sometimes they could not accommodate the additional time to educate and enroll patients. A provider commented that they “underappreciated the coaching that the patients require at the time of enrolling.” There was a tendency for providers to view the enrollment process as not in their scope of work and as a task more suited for nurses or support staff, suggesting that there be, “someone [nonprovider] assigned to assist with Annie.”

Notwithstanding difficulties incorporating the aTS into established clinical practices, providers believed the aTS was welcomed by patients (7/10, 70%), could enable clinics to meet patient-centered care goals (9/10, 90%), and could lead to cost savings for the VA (6/10, 60%). Most providers intended to continue using the aTS for HCV (9/10, 90%) and would use it for other health conditions after study completion (8/10, 80%).

## Discussion

### Principal Findings

To our knowledge, this is this first randomized evaluation of implementation facilitation strategies intended to increase adoption of VA’s aTS. We found that patients and providers largely accepted and deemed the aTS appropriate, easy to use, and useful, albeit with substantial barriers to uptake and sustained use. Greater patient recruitment by providers was associated with more facilitation and lower baseline satisfaction with clinic HCV care processes. We found differences in implementation outcomes. For some of the stages of aTS implementation, patient engagement was higher in AI (vs UI). Overall, 1 out of 6 patients (16%) who were offered the aTS received aTS text messages. Texters, compared with nontexters, felt more connected to their care team, confident in their HCV medication taking, and more activated for self-management. There were no differences, however, in clinical effectiveness outcomes between patients based on use of the aTS system (texters vs nontexters). Our results suggest that the aTS may not be easy to implement but has better chances of success if several PRISM domains are attended to at the adoption, implementation, and sustainability phases.

### Augmented Implementation Facilitation

Within the implementation and sustainability infrastructure domain, a need for facilitated implementation was identified. Considerable effort was needed to assist all clinics with aTS adoption. Once implementation began, the aTS called for episodic technical assistance rather than high-intensity, sustained facilitation [35]. More facilitator-provider interaction appeared to be related to higher aTS recruitment, as others have demonstrated [36]. We also found that facilitation may have replaced the need for a toolkit in this study because the facilitator was highly accessible to providers. There is mounting evidence that toolkits and manuals tend to be underused when other implementation strategies are available [37,38]. This may be due to toolkit development being unstandardized and thus highly variable. For this reason, Hempel and colleagues [39] recently provided recommendations for the content, development, and evaluation of quality improvement toolkits. Of the implementation facilitation meta-strategies in this study, it appeared site visits had a strong influence on implementation, suggesting that the interpersonal component of a facilitator-provider dyad is paramount to successful implementation.

Our results also indicate that despite differences in planned facilitation approaches, there were no differences in the dose of facilitation delivered, suggesting facilitation efficiency is enhanced when it is delivered in person or is channeled through a champion (as with augmented implementation sites) [40]. Our findings are consistent with current literature that facilitator effort tapers once clinics have commenced implementation [37,41]. Facilitation, in many ways, is not formulaic and is inherently dependent on local context and need. Notably, in our work, a common implementation strategy was not included—there was no explicit benchmarking or audit and feedback component to the facilitation efforts. More guidelines on how to gauge specific facilitation need (ie, activities, dose, intensity, timing of start, and removal) are necessary, as are ways to track, evaluate, and replicate with fidelity.

### Assessing Readiness to Implement

Taking an organizational perspective to understand both the intervention and its recipients illuminates several important factors that can influence aTS uptake. Organizational readiness between clinics differed in only one area: satisfaction with local HCV care practices. Providers who were unsatisfied with their HCV care practices may have perceived their clinics as needing improvement and thus were more likely to embrace the aTS. As such, some clinics were primed for the introduction of a new practice, despite no mandate or clinical practice guideline motivation. Once providers began implementing, however, several believed the aTS did not integrate well into their workflow and was a more fitting task for nonclinicians. Insights from organizational readiness assessments tend to be underused but may help understand facilitation mechanisms of action, which likewise remain poorly understood and often unmeasured [42].

We also found variation in the assumptions made by providers regarding patient candidacy for aTS recruitment. UI clinics registered and enrolled more patients; however, AI clinic patients authenticated their phone numbers at higher rates thus triggering the text messages to start. Providers at AI clinics may have been targeting patients more selectively, thus making their recruitment more efficient. While potentially successful at their AI clinics, in general, providers should be wary of the validity of their selection heuristics, which studies indicate are often unreliable [43]. It may be more appropriate to offer the aTS universally to patients rather than selectively recruiting those deemed more apt to agree, thereby reducing proficient user bias. Future studies could explore alternative methods for introducing the aTS and consenting and registering patients, such as through patient opt-out and self-enrollment approaches. Since study completion, adjustments to the aTS registration procedure now allow for support staff to register patients in the aTS portal on behalf of a consenting clinician.

### Patient Behavior Change

There were several facilitating patient perspective elements within the intervention and recipients domains important to aTS use and sustainability. There was near universal positive feedback from patients about the ease of use and benefits of the aTS, however there were notable drop-offs in engagement during phone number authentication due to syntax errors and possible changes in willingness to use. After interacting with the aTS, texters compared with nontexters reported feeling more activated for self-management and adherence to medication and improved health status, but no significant differences in clinical outcomes were detected. As the transtheoretical model posits and as the aTS begins to show, attitudinal changes precede behavior change and may produce meaningful behavior change [44]. Because polypharmacy among veterans is common, additional work is required to understand the types of patients most likely to benefit from texting interventions [45-47]. There may be other important patient moderating variables, such as age and rurality, that determine whether individuals choose to adopt the aTS [48]. While communication preferences are shifting in favor of texting and other virtual modalities, slower-than-anticipated uptake of technology tools for health care is common [49]. Nonetheless, texting differs from other health technologies in that it reaches patients where they are, at any time, and can accept responses whenever patients are ready to offer them. In depth qualitative work is needed to understand barriers to implementation and sustained use of the aTS and how to redesign the enrollment and engagement process.

### Strengths and Limitations

This was the first study of VA’s aTS. Strengths of this study are that PRISM was used to guide the evaluation, it was a randomized design, and we simultaneously studied implementation and effectiveness outcomes using mixed methods. We believe this study makes an important contribution to advancing the implementation science of texting interventions.

Our study has several potential limitations. First, our study was conducted within the VA and for the treatment of HCV; therefore, not all settings or health behaviors were represented, which limits our generalizability. Second, we selected clinics that expressed interest in using the aTS, raising the potential for selection bias. Third, the sicker, more socially and psychologically vulnerable patients may not have been invited by providers to participate in the aTS because of concerns that it could be confusing or costly for them. This selection bias may explain some of the apparent beneficial findings for texters. Also, feedback on the aTS may have been subject to social desirability bias. We did not adjust for multiple comparisons due to the explanatory nature of the study.

### Conclusions

Increasingly, health care systems are using technology to meet patient expectations for electronic transactions, information exchange, and on-demand access to providers. This was the first study to examine the implementation and effectiveness of an automated text messaging system in the VA generally and for HCV treatment specifically. Despite positive perceptions of the aTS, patient enrollment was challenging; however, augmented facilitation resulted in greater sustained engagement of patients once they enrolled. Importantly, among patients who used the aTS (the texters) there was an indication of improved illness perception, health engagement, and patient activation. Our results suggest that the aTS can serve as an adjunct tool to usual HCV care, provided it is appropriately integrated into clinical workflow. Our study has implications for health care systems making efforts to engage patients beyond episodic in-person visits through patient-facing technologies. Findings suggest that a large pool of potential texting adopters have yet to realize benefits from this technology. While novel technologies such as the aTS have considerable potential, they also present distinct behavioral, social, and technical challenges for implementation and scale-up.

## Abbreviations

AI: augmented implementation
aTS: automated text messaging system
HCV: hepatitis C virus
NHS: National Health Service
PRISM: Practical, Robust Implementation and Sustainability Model
SMS: short message service
SVR: sustained virologic response
UI: usual implementation
VA: Department of Veterans Affairs
